# The effect of text and graphic cue-based action observation on the working memory performance of novice badminton players

**DOI:** 10.3389/fpsyg.2024.1439528

**Published:** 2025-01-06

**Authors:** Dukchan Jang, Junho Ha

**Affiliations:** ^1^Department of Physical Education, Keimyung University, Daegu, Republic of Korea; ^2^Department of Practical Physical Education, Keimyung College University, Daegu, Republic of Korea

**Keywords:** action observation, visual cues, cognitive information processing, working memory, badminton

## Abstract

**Introduction:**

Understanding strategic situations is essential in sports. There has been relatively little research examining the effectiveness of action observation based on visual cues in strategic situations. This study investigated whether action observation with visual cues can help performers understand the strategic aspects of complex sports by analyzing the effect of text cue-based action observation and graphic cue-based action observation on the accuracy and speed of cognitive information processing in working memory.

**Methods:**

Forty-four male and female novice badminton players participated in the experiment. They were randomly assigned to one of four groups: text cue-based action observation (TAO), graphic cue-based action observation (GAO), action observation (AO), and a control group (CON). The experimental design consisted of a pre-test, intervention, and post-test. The experiment analyzed the accuracy and response time of cognitive information processing in working memory.

**Results:**

The accuracy and response time analysis showed that the AO group significantly improved their cognitive performance accuracy and response time from pre-test to post-test compared to the control group. The TAO and GAO groups with visual cues significantly outperformed the AO and CON groups for accuracy. However, only the TAO group significantly outperformed the other groups in term of response time. The GAO group improved significantly compared to the CON group but not significantly compared to the AO.

**Conclusion:**

These results suggest that visual cues can influence the modulation of cognitive load in working memory and that TAO is a relatively more efficient perceptual-cognitive training method for novices.

## 1 Introduction

Skilled performers have superior perceptual-cognitive abilities and motor performance compared to novices (Del Villar et al., 2007; Kim et al., 2019; Ste-Maire, 1999; Thomas and Thomas, 1994). This implies a potential correlation between perceptual-cognitive ability and motor performance (Carroll and Bandura, 1987). Learning a motor skill results in simultaneous improvements in both physical skills and perceptual-cognitive abilities through the physical repetition of that skill (Coker, 2021; Fitts and Posner, 1967). However, motor skills can also be practiced repeatedly and covertly without overt physical practice, and one of the most common cognitive training methods for this is action observation. Action observation has contributed to the formation of sophisticated mental representations of functional aspects of motor skills (Frank et al., 2018; Kim et al., 2017) and the acquisition of physical skills (Kernodle and Carlton, 1992; Kernodle et al., 2001; Laguna, 2008; Scully and Carnegie, 1998) based on perceptual-cognitive processes without actual physical action. However, in motor learning, it is essential to not only improve physical skill proficiency but also enhance one’s understanding of the strategic context. This deeper comprehension allows learners to make more informed decisions and adapt their skills to dynamic and varied situations. To achieve more successful motor performance, it is necessary to examine whether action observation, based on perceptual-cognitive processes, can contribute to the improvement of decision-making skills by enhancing strategic knowledge, much like cognitive training methods improve physical skills.

While extensive research (Frank et al., 2018; Kernodle and Carlton, 1992; Kernodle et al., 2001; Laguna, 2008) has explored the application of cognitive training methods for improving physical skills, there has been very little investigation into its effectiveness for enhancing strategic understanding. In traditional sports settings, strategic understanding processes are typically learned implicitly alongside the development of motor skills. However, the nature of some tasks, such as predicting the trajectory of the shuttlecock in badminton based on its speed, angle, and spin or identifying tactical patterns during a rally, suggests that focused observation-based cognitive training could lead to more efficient organization of strategic understanding and the formation of detailed mental representations. Acquiring strategic knowledge can facilitate information processing and enable rapid adaptation to the target behavior, especially under specific environmental demands (Hibbs and O’Donoghue, 2013). Therefore, it is essential to acquire core strategic knowledge in various sports situations. To effectively apply strategic knowledge to novices in the context of motor learning, it is essential to consider the learning stage model.

According to the learning stages model, it is argued that the learning of motor skills progresses through three distinct stages (Fitts and Posner, 1967). In the first stage, the cognitive stage, the novice performer must attend to cognitive issues related to what to do, significantly increasing cognitive load in working memory. At this stage, working memory is required to process and integrate information about the task, which is often overloaded as the learner is consciously aware of every action and decision. In the second, associative stage, the demand on working memory capacity is gradually reduced as the association between specific cognitive stimuli and the movement required to achieve skill goals is progressively strengthened. This reduction in load occurs because the learner starts to form more efficient memory representations, making the cognitive process more automatic and less reliant on working memory. In the final stage, called the automatic stage, the cognitive load on working memory is minimal, as the learner can perform motor skills without conscious thought. At this point, the task becomes so well-practiced that it is stored in long-term memory, and working memory is used minimally for routine movements. Therefore, working memory plays a crucial role in processing and managing cognitive load during the initial stages of motor skill learning, while its involvement decreases as the learner becomes more proficient.

In the early stages of motor skill learning, the repetitive processing of information within working memory facilitates the transfer of knowledge to long-term memory. However, working memory serves not only to transfer information through repeated demonstration but also as a temporary workspace that integrates recently presented information with information retrieved from long-term memory (Cowan, 2008; Diamond, 2013). Unlike experienced experts, novices do not have explicit representations of knowledge in long-term memory related to the task. This makes it challenging to solve problems, make decisions, and prepare for tasks requiring multiple decisions in sports, as novices struggle to integrate information from long-term memory with new, presented information (Baddeley, 1986; Baddeley and Hitch, 1974). However, action observation may only partially depend on mental representations in long-term memory, as it is externally guided by extrinsic stimuli such as demonstrations or recorded videos and is completed in a perceptual-based process (Holmes and Calmels, 2008; Vogt et al., 2013). In other words, externally presented visual information is processed in working memory and does not necessarily rely on representations stored in long-term memory (Kim et al., 2017). For example, a recent study by Frank et al. (2018) showed that action observation training for novice golfers helps to build more elaborate mental representation structures with improved performance. These findings suggest that even when applied early, before a knowledge base is established, action observation training can enhance the functional adaptation of mental representations for specific tasks. Given the results reviewed so far, action observation with strategic knowledge may also be suitable as a cognitive training tool for novices. However, traditional action observation protocols often contain many distractors, which may overload the working memory system of novice learners, who need to comprehend both physical activity and strategic knowledge simultaneously.

Bandura’s (1971) social cognitive theory highlights the critical role of attention, along with retention, reproduction, and motivation, in observational learning (i.e., modeling). Particularly, attention and retention are essential cognitive steps for learning to occur effectively, as they enable the learner to focus on and internalize observed actions (Laguna, 2008). For these processes to function efficiently, the use of cues may be required to guide and support cognitive engagement. Observational learning methods that incorporate visual cues can enhance cognitive processing by directing attention to essential aspects of the task (Boucheix and Lowe, 2010; D’Innocenzo et al., 2016; Horvath, 2014; van Gog, 2014), reducing inefficient mental activity, and free up working memory resources. Novices, who rely heavily on bottom-up processing and often experience cognitive overload due to high-frequency visual exploration, can particularly benefit from visual cues that guide their attention to essential elements of action observation material or emphasize critical idea (D’Innocenzo et al., 2016; Horvath, 2014; van Gog, 2014). This approach could provide a stepping stone to learning how to read matches (Fortin-Guichard et al., 2020).

Despite these potential benefits of visual cues in observational learning, existing studies present mixed results regarding their impact, particularly in the domain of motor skill acquisition. These inconsistencies appear to stem from various methodological differences, such as task characteristics, the learner’s experience level, and how observational learning is structured and presented. Nevertheless, a notable takeaway from prior research is that the presence of attention cues significantly enhances performance and learning outcomes. For example, less than 30% of reviewed studies without cues showed positive results, whereas over 60% of studies incorporating cues demonstrated significant improvements (Mödinger et al., 2022; Rothstein and Arnold, 1976). These findings highlight the importance of using cues to reduce uncertainty, enhance motion detection, and guide learners’ attention toward task-relevant aspects (Ball and Sekuler, 1981). Building on these findings, studies exploring the use of verbal cues in observational learning provide further insights into how targeted instructions can impact motor skill acquisition. For example, Kernodle and Carlton (1992) required participants to observe video replays of their just-complete performance while providing verbal cues beforehand. These verbal cues guided participants on where to focus their attention or what to do to improve throwing distance in subsequent attempts. The group receiving cues demonstrated more effective acquisition of throwing form compared to groups receiving only knowledge of results (KR) or knowledge of performance (KP). Similarly, another study required participants to engage in observational learning with verbal error-correction feedback cues and verbal cues on how to improve in subsequent trials (Kernodle et al., 2001). Interestingly, the verbal cue group outperformed the observational learning with verbal error-correction feedback cues group in a retention test, showing greater improvements in throwing distance and form. The findings of these studies suggest that the effects of cues in observational learning may vary depending on their relevance to the observational context. However, a limitation of these studies is that they did not directly incorporate visual cue information into the observational materials, making it unclear whether the observed effects were due to the verbal feedback cues or the observational learning process itself. This ambiguity dilutes a clear understanding of the specific effects of visual cues in observational learning, underscoring the need for further research to directly explore the impact of embedded visual cues on cognitive processes.

Actually, a study has examined the relationship between action observation, cognitive processing, and motor sill acquisition, with a focus on the accuracy of cognitive representations based on long-term memory (Laguna, 2008). However, this study does not incorporate inherent visual cues with the action observation itself either. On the other hand, we distinguish between text-based and graphic-based visual cues embedded within action observation materials and focus on how providing these cues influences cognitive processing in working memory during action observation tasks. Based on studies that have applied visual cues to improve learner understanding in animations (Boucheix and Lowe, 2010; De Koning et al., 2007; Mayer and Moreno, 2003), these cues can be categorized into text-based and graphic-based visual cues (Mayer, 2009). Text-based cues use words or sentences to highlight critical information (Hayes and Reinking, 1991; Xie et al., 2019), while graphic-based cues employ visual elements like arrows, highlights, or zooming to draw attention to essential details (Lin and Atkinson, 2011; Jamet et al., 2008; Jeung et al., 1997). By enhancing voluntary attention and reducing distractions, visual cues learners to process information more deeply, integrate it effectively with prior knowledge in working memory, and improve their understanding of the relationship between attention and observation. These cues also help avoid overloading working memory, which is particularly critical for novices who may struggle to process transient information without structured guidance (Brockhoff et al., 2022).

Building on this understanding, it is crucial to design action observation effectively to address the issue of transient information processing effects and cognitive overload in working memory. To achieve this, observational materials with visual cues embedded directly with the action observation itself were used, and these cues conveyed content that held significant meaning for task performance. In this study, based on the previous review, we hypothesized that if visual cue-based action observation (i.e., text, graphic) can increase information processing efficiency in working memory compared to mere action observation from a cognitive load perspective, then cognitive information processing accuracy and speed in working memory would be efficiently improved through short-term treatment. In addition, we hypothesized that a group of text-based visual guides using keywords may perform better than a group of graphic-based visual guides because text cue-based action observation can more specifically emphasize the structure and relationships between elements necessary for understanding the task during action observation for novices with limited knowledge.

This study aimed to determine whether action observation with visual cues can help understand the strategic aspects of complex sports by analyzing the effect of text cue-based action observation and graphic cue-based action observation on the accuracy and speed of cognitive information processing in working memory.

## 2 Methods

### 2.1 Participants

Forty-four male and female novices (male = 26, female = 18) aged 18–25 (*M*_age_ = 21.23, *SD* = 1.61) with little badminton experience participated in the experiment (see Table 1). They were assigned to one of four groups in a random manner with eleven participants in each group: text-based visual guidance cues AO group (*M*_age_ = 21.00, *SD* = 1.41), graphic-based visual guidance cues AO group (*M*_age_ = 21.10, *SD* = 2.02), AO group (*M*_age_ = 21.00, *SD* = 1.56), and CON group (*M*_age_ = 21.80, *SD* = 1.48). All participants were right-handed and had normal or corrected-to-normal vision. Participants had less than one month of badminton experience, and their experience was checked before participating in the experiment through self-report. Participants volunteered for the experiment, were provided with detailed information about the study, and gave written informed consent before participation. They reported through self-reports that they were healthy and had no recent cognitive or neurological problems. Recruitment was conducted through a participant recruitment announcement provided by researchers. Participants were offered a small cash incentive (15,000 KRW, approximately 11 USD). The study was reviewed and approved by the Institutional Review Board and conducted in accordance with the ethical standards stated in the 1964 Declaration of Helsinki.

**Table 1:** Participants’ demographic information and baseline memory ability.

Category	Text	Graphic	Action	Control	*F*	*p*
Age	21.00 ± 1.41	21.10 ± 2.02	21.00 ± 1.56	21.80 ± 1.48		
Gender (female/male)	4/7	4/7	5/6	5/6		
Memory ability (accuracy)	66.81 ± 8.49	63.87 ± 15.52	61.25 ± 10.12	61.58 ± 9.44	0.34	0.80
Memory ability (speed)	4559.22 ± 1567.74	4629.60 ± 1354.61	4836.63 ± 707.71	5279.82 ± 1350.27	1.47	0.24

No significant differences in memory ability between groups at baseline. Text = Text cue-based action observation; Graphic = Graphic cue-based action observation; Action = Action observation. Memory ability (accuracy): Percentage of correct responses out of 48 scenes, reflecting the ability to correctly identify the appropriate doubles formation; Memory ability (speed): Response time in milliseconds, reflecting the speed of processing and decision-making in each scene. Mean ± SD = Mean and standard deviation.

### 2.2 Measurement

#### 2.2.1 Memory assessment

A memory ability evaluation program was applied to evaluate the general memory ability of the participants as an individual variable that can affect cognitive information processing ability in working memory (Robinson and Brewer, 2020; Rockstroh and Schweizer, 2001). The program for evaluating memory ability was developed for the experiment using OpenSesame (Mathôt et al., 2012), an open-source program. Initially, 30 short everyday words were presented simultaneously on the monitor for 2 min, and participants were asked to memorize as many of the presented words as possible. Afterward, to evaluate memory ability, 60 words (i.e., 50% previously presented words, 50% new words) were presented one at a time in random order on the monitor. Participants were instructed to press button one on the keyboard if the word had been included in the words shown in the initial 2 min. If the word was not previously presented, they had to press button two. Participants were instructed to respond to each word as accurately and quickly as possible. If participant responded correctly to all 60 presented words, they received a score of 100%. However, accuracy decreased with each incorrect response to the words. Both accuracy and response time were included to assess not only the correctness of memory recall but also the speed of cognitive processing, offering a more comprehensive measure of memory function. The accuracy and response time for the participant’s judgment for each word presented were automatically stored in the computer and analyzed after the experiment.

#### 2.2.2 Mental effort

To measure cognitive load, Paas’s (1992) reliable and valid mental effort scale, a single-item measurement tool, was applied. Participants rated the level of mental effort they invested in completing the task by responding to the question, “How much mental effort did you invest during the task?” on a 7-point Likert scale ranging from (1) “very low” to (7) “very high.” Responses were recorded on a single sheet of paper by checking the appropriate box. Higher scores indicate that participants perceived the task as requiring more mental effort, providing an effective and straightforward assessment of cognitive load.

#### 2.2.3 Task difficulty

Task difficulty was assessed using a 7-point Likert scale to capture participants’ perceived difficulty in understanding and performing the task. Participants responded to the question, “How difficult was it to perform the task?” by selecting their response on a single sheet of paper, with responses ranging from (1) “very easy” to (7) “very difficult.” Higher scores indicate greater perceived difficulty in both understanding and executing the task, providing an overall indication of task challenge.

#### 2.2.4 Cognitive performance

In this study, the working memory test developed with OpenSesame (Mathôt et al., 2012) was used to measure the accuracy and speed of the information processing in the doubles formation related to various badminton techniques in working memory. OpenSesame, often used in psychology and cognitive science research, provides an intuitive interface for designing and conducting diverse experiments. In this study, it was used to evaluate participant’s working memory abilities. By combining stimuli such as text and images, a working memory task was created to focus on measuring the ability to temporarily store and manipulate specific information.

One of eight badminton techniques (i.e., long serve, high clear, underarm clear, short serve, drop, hairpin, smash, drive) was randomly displayed on the computer monitor. For each skill, six different activity scenes were randomly presented on the screen, one at a time, totaling 48 scenes. Accuracy was recorded based on whether participants responded correctly to the given activity scene. Specifically, if the shuttlecock rose in the opponent’s court, our team adopted a side-by-side defensive formation to more effectively cover space potential smashes. Alternatively, when the shuttlecock dropped in the opponent’s court, our team assumed a top-and-back formation, preparing for a more offensive stance, as the shuttlecock was likely to be returned high, offering an attacking opportunity. Response time was measured from the moment each paused scene was presented until the participant pressed a button. In each badminton doubles scenario, participants were shown an action observation scene that had been paused and were asked to respond to each badminton activity scene. Each scene remained on the screen until a participant responded, and immediately after their response, the next scene was displayed. The participant’s task was to determine the correct doubles formation in the paused action observation just before the opponent’s return while our team’s shuttlecock was flying toward the opponent. Participants were asked to determine, as accurately and quickly as possible, whether our team’s doubles formation would be better, as either top-and-back (i.e., keyboard button one was pressed) or side-by-side (i.e., keyboard button two was pressed) after the opponent’s return. Furthermore, OpenSesame’s data collection and analysis features were used to record accuracy and response time with high precision. The collected data was then exported in CSV format for further statistical analysis in specialized software. The accuracy and response time for each participant’s judgment were stored separately in the computer.

### 2.3 Intervention

**Figure 1:**
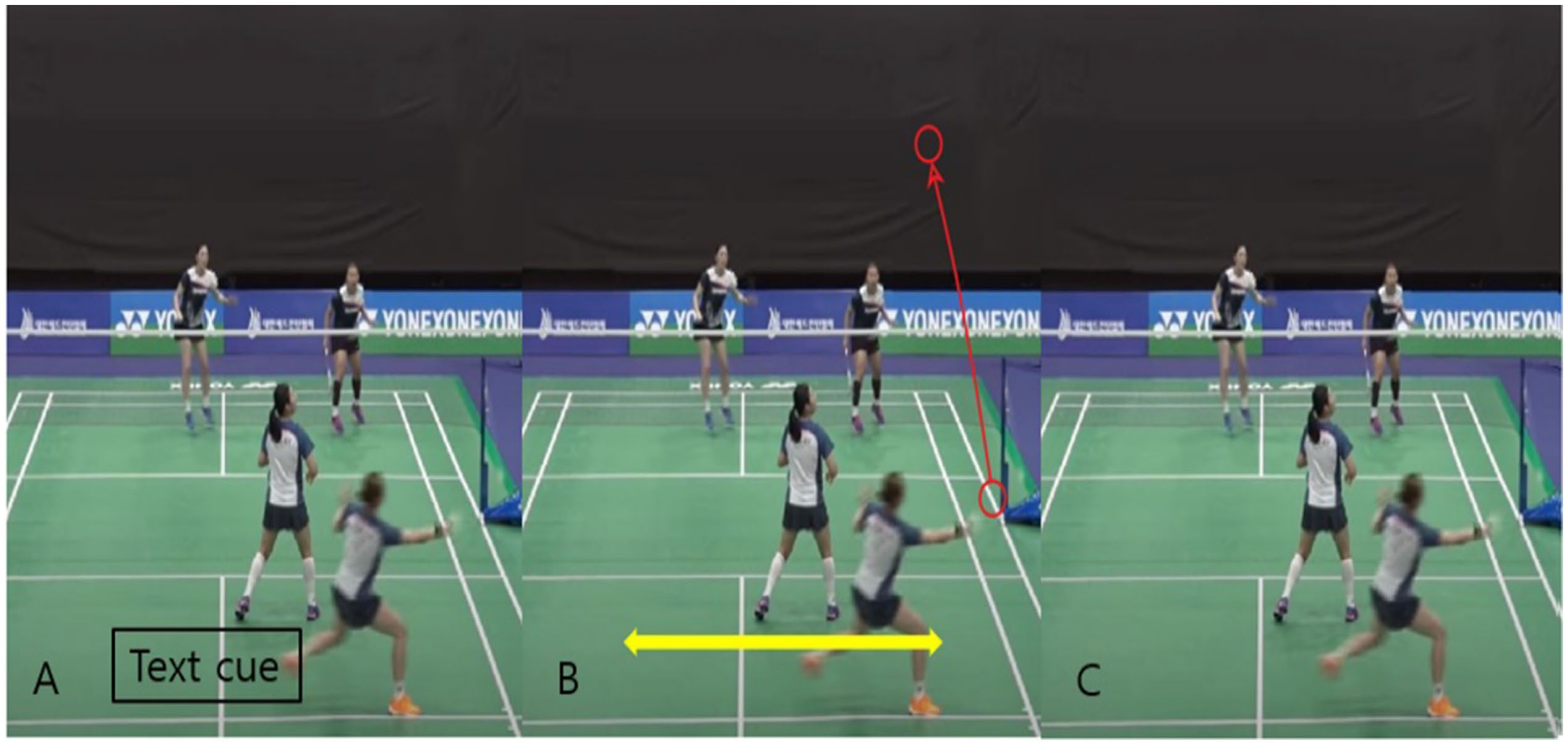
Visual representation of intervention types: **(A)** text cue-based action observation, where text cues are displayed in response to shuttlecock movement (e.g., if the shuttle floats up, the best position is side-by-side); **(B)** graphic cue-based action observation, with symbolic arrows indicating correct positioning. Thin arrows represent the trajectory of the rising shuttlecock, while bold arrows indicate the side-by-side positioning instruction; and **(C)** action observation without cues. The scenes shown represent examples of group-specific responses to the high clear badminton skill.

After the pre-test, participants were divided into different intervention groups, each of which received a specific form of action observation to guide their decisions about the correct doubles formation (see Figure 1). For the text cue-based action observation group, participants watched scenes in which our team’s shuttlecock flew toward the opponent’s side. In scenes where the shuttlecock was rising, a text cue, “if the shuttle floats up, the best position is side-by-side,” appeared at the bottom of the screen. In scenes where the shuttlecock was descending, the text cue changed to “if the shuttle goes down, the best position is top-and-back.” This group did not receive any additional verbal cues regarding the opponent’s movements. The graphic cue-based action observation group viewed the same action scenes, but instead of text, symbolic graphic cues were displayed at the end of each scene. Left and right arrows indicated the “side-by-side” position, and up and down arrows represented the “top-and-back” position. Participants in the action observation group watched the same action scenes as the previous groups, but no cues-either text or graphic-were provided regarding the doubles formation. This group relied on the visual observation of the action scene alone to determine the best formation. The control group did not receive any action observation related to badminton. Instead, they watched unrelated videos for the same duration as the other groups.

Each participant in all groups observed 16 scenes, with each of the 8 badminton skills presented twice. The action observation training program allowed participants to view the scenes at their own pace. The scenes progressed only when the participant pressed a key on the keyboard, and the total duration of the intervention was approximately 15 ~ 20 min. After completing the intervention, participants were given 3 min to fill out a self-report questionnaire assessing their perceived mental effort and task difficulty. Following a 15-min break, participants underwent the post-test (i.e., cognitive performance), which followed the same procedure as the pre-test.

### 2.4 Procedure

Participants visited the laboratory individually during the experiment, and they signed a consent form for the experiment after receiving a sufficient explanation. The experimental design consisted of a pre-test, intervention, and post-test. In the pre-test, the evaluation program for memory ability was used to measure the participant’s general memory ability, an individual variable affecting cognitive performance in working memory (Robinson and Brewer, 2020; Rockstroh and Schweizer, 2001). The participant sat comfortably in a chair with armrests and stared at the monitor (display size 32 inches, resolution 3,840 × 2,160, the distance between the participant and monitor 50 cm). A red dot was temporarily presented on the white screen of the monitor to indicate readiness for measurement of general memory. Then, 30 common words were presented on the monitor for 2 min. Participants were asked to memorize as many words as possible for 2 min. After 2 min, 60 common words, including the previously presented words, were presented one by one in random order, and the participant responded only to the previously presented words as accurately and quickly as possible (i.e., press keyboard button one for the previous 30 words; press keyboard button two for the other). After completing the memory test, participants proceeded to the working memory test, which was designed to assess cognitive performance in a badminton doubles context. A paused badminton action observation scene was presented after a red dot was temporarily displayed on a white screen. Participants were asked to judge the formation of the correct doubles for each action observation. The action observation presented to the participants was a scene in which our team’s shuttlecock flew to the opposing team in the badminton doubles situation. The paused scene was just before the opposing team’s return. Participants were asked to judge which formation (i.e., side-by-side, top-and-back) was more appropriate for the scene. Scenes representing six different badminton techniques were used in the experiment. A total of 48 scenes were presented in random order, and participants were instructed to decide which formation (i.e., side-by-side, top-and-back) was more appropriate for each scene, responding as accurately and quickly as possible.

After the pre-test, the intervention was conducted according to the procedures outlined above. Following the intervention, a post-test (i.e., cognitive performance) was administered, which was carried out in the same manner as the pre-test. The entire experimental process was recorded to ensure the preservation of data.

### 2.5 Data analysis

#### 2.5.1 Memory ability evaluation

To analyze general memory ability, accuracy and response time data collected from the OpenSesame (Mathôt et al., 2012) program were processed. Accuracy was defined as the percentage of correctly identified words out of the total 60 presented words, with a maximum score of 100%. Response time represented the average time taken by participants to respond to each word. The data from individual trials were averaged across participants to produce group-level measures for statistical comparisons.

#### 2.5.2 Mental effort

For data analysis, the mental effort scores from the 7-point Likert scale were aggregated by calculating the mean value across participants in each group. These mean values were used to compare cognitive load between groups. This approach allowed for group-level interpretation of the perceived mental effort invested in the tasks.

#### 2.5.3 Task difficulty

To understand perceived task difficulty, participants’ ratings on the 7-point Likert scale were averaged across participants in each group to calculate group-level mean scores. These mean scores provided a basis for comparing perceived task difficulty between groups. This approach facilitated an aggregated interpretation of participants’ perceptions regarding the challenge of understanding and performing task.

#### 2.5.4 Cognitive performance in working memory

Cognitive performance was evaluated using two dependent variables: accuracy and response time. Accuracy was calculated as the percentage of correct responses out of the total 48 scenes, providing a measure of participants’ ability to correctly identify the appropriate double formation. Response time, measured in milliseconds, reflected the speed at which participants processed and responded to each paused scene. For each participant, accuracy and response time data were averaged to compute mean values, enabling comparisons across groups. These aggregated measures provided insights into the cognitive processing efficiency in working memory under the experimental conditions.

### 2.6 Statistical analysis

General memory ability, mental effort, and task difficulty variables were measured once for all groups. Therefore, four groups (text cue-based AO, graphic cue-based AO, AO, CON) were subjected to a one-way analysis of variance (ANOVA) to assess between-group differences. For working memory, since measurements were taken at two time points (pre-test and post-test) across the four groups, a mixed-design was employed. A two-way factorial ANOVA with repeated measures on the second factor was used to analyze the effect of the intervention on cognitive performance. The factors in this analysis were the group (4 levels: text cue-based AO, graphic cue-based AO, AO, CON) and test session (pre-test, post-test). Significant main and interaction effects were followed with Bonferroni-corrected *post hoc* and simple t-tests. Mean differences are reported. All data analyses were conducted using IBM SPSS Statistics 28.0, with an alpha level set at 0.05 to determine statistical significance.

## 3 Results

### 3.1 Memory ability

Memory ability, which represents the general memory ability of the participants as an individual difference variable, was divided into accuracy and response time. When analyzing the accuracy of general memory ability, there was no significant main effect for group [*F*(3,40) = 0.34, *p* = 0.80, $\eta_{p}^{2}$ = 0.03]. There was also no significant main effect for group [*F*(3,40) = 1.47, *p* = 0.24, $\eta_{p}^{2}$ = 0.10] in the response time for general memory ability (see Table 1).

### 3.2 Mental effort

As a result of analyzing the mental effort, an index of cognitive load investment for the task, there was no significant main effect for group [*F*(3,40) = 0.26, *p* = 0.86, $\eta_{p}^{2}$ = 0.02] (see Table 2).

**Table 2:** Comparison of mental effort and task difficulty by group.

Measure	Text	Graphic	Action	Control	*F*	*p*
Mental effort	6.40 ± 0.69	6.50 ± 0.84	6.10 ± 0.87	6.00 ± 0.81	0.26	0.86
Task difficulty	2.90 ± 1.28	3.00 ± 1.24	3.70 ± 0.99	3.30 ± 1.15	1.12	0.35

No significant differences in mental effort and task difficulty between groups. Text = Text cue-based action observation; Graphic = Graphic cue-based action observation; Action = Action observation. Mean ± SD = Mean and standard deviation.

### 3.3 Task difficulty

As a result of analyzing the perceived task difficulty score, there was no significant main effect for group [*F*(3,40) = 1.12, *p* = 0.35, $\eta_{p}^{2}$ = 0.08] (see Table 2).

### 3.4 Cognitive performance in working memory

#### 3.4.1 Accuracy

**Figure 2:**
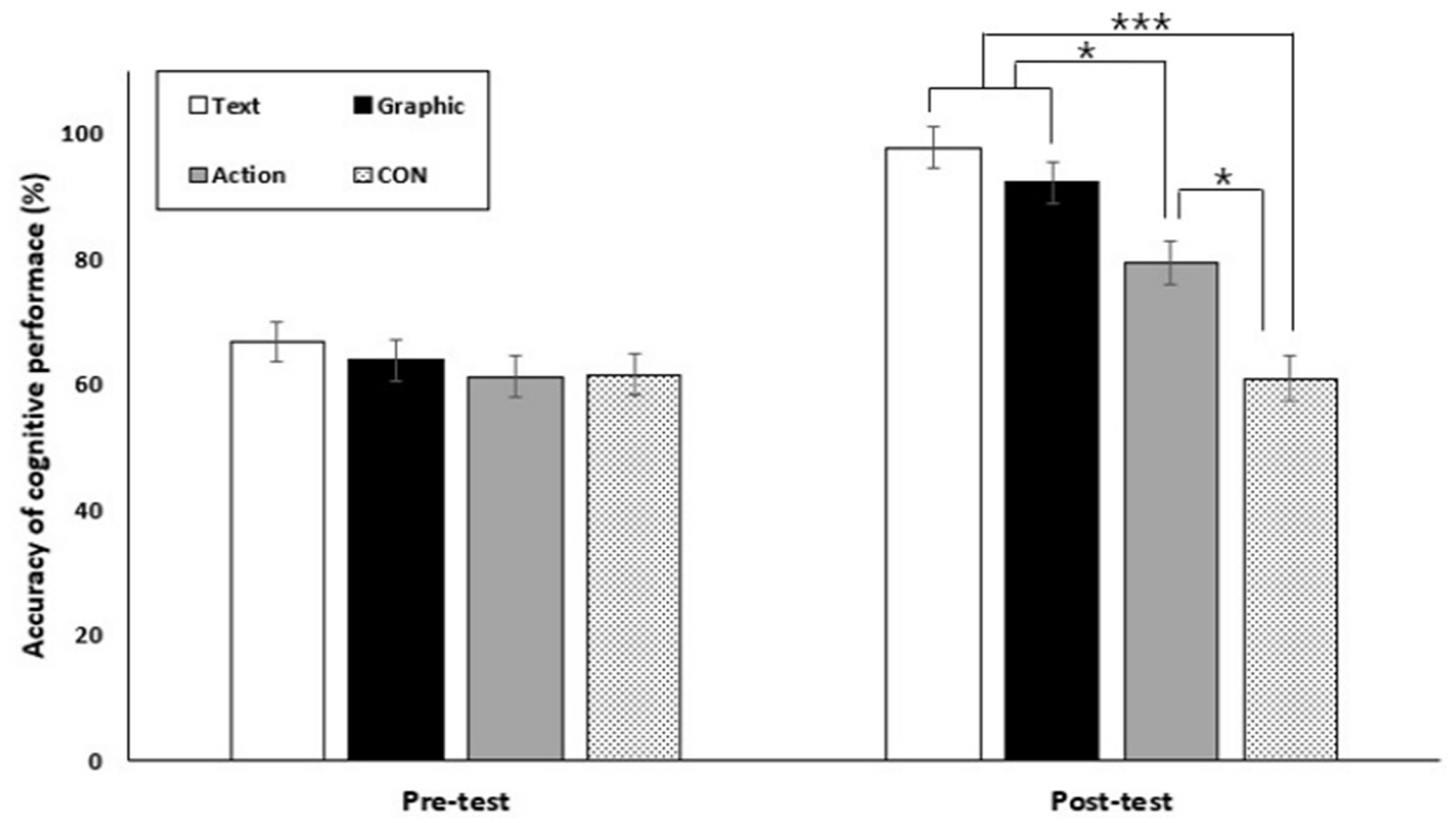
Accuracy of cognitive performance in working memory for text cue-based action observation (Text), graphic cue-based action observation (Graphic), action observation (Action), and control groups (Control) across pre-test and post-test conditions. Error bars represent the standard deviation of the group means. The asterisks indicate significant differences. ^*^p < 0.05, ^***^p < 0.001.

The analysis of the accuracy of cognitive performance in working memory revealed that the main effects of group [*F*(3,40) = 13.58, *p* < 0.001, $\eta_{p}^{2}$ = 0.51] and test session [*F*(1,40) = 83.14, *p* < 0.001, $\eta_{p}^{2}$ = 0.68] were significant, respectively. The result of the *post hoc* test on the main effect of group showed that the text cue-based AO group performed significantly better than the AO group (*p* < 0.01) and the control group (*p* < 0.001). The graphic cue-based AO group was significantly better than the control group (*p* < 0.001). The result of the post hoc test on the main effect of test session showed that the accuracy of the post-test was significantly better than that of the pre-test (*p* < 0.001). The interaction effect of group and test session was also significant [*F*(3,40) = 11.62, *p* < 0.001, $\eta_{p}^{2}$ = 0.47] (see Figure 2). *Post hoc* comparisons by way of paired t-tests revealed that the text cue-based AO group (*p* < 0.001), the graphic cue-based AO group (*p* < 0.001), and the AO (*p* < 0.05) performed significantly better in the post-test than the pre-test. However, there was no significant difference between the pre-test and the post-test in the control group (*p* = 0.86). *Post hoc* comparisons by way of one-way ANOVA revealed no significant difference between groups in the pre-test (*p > 0*.05). However, in the post-test, the text cue-based AO group and graphic cue-based AO group performed significantly better than the AO and control group (*p* < 0.05). The AO group was significantly better than the control group (*p* < 0.01).

#### 3.4.2 Response time

**Figure 3:**
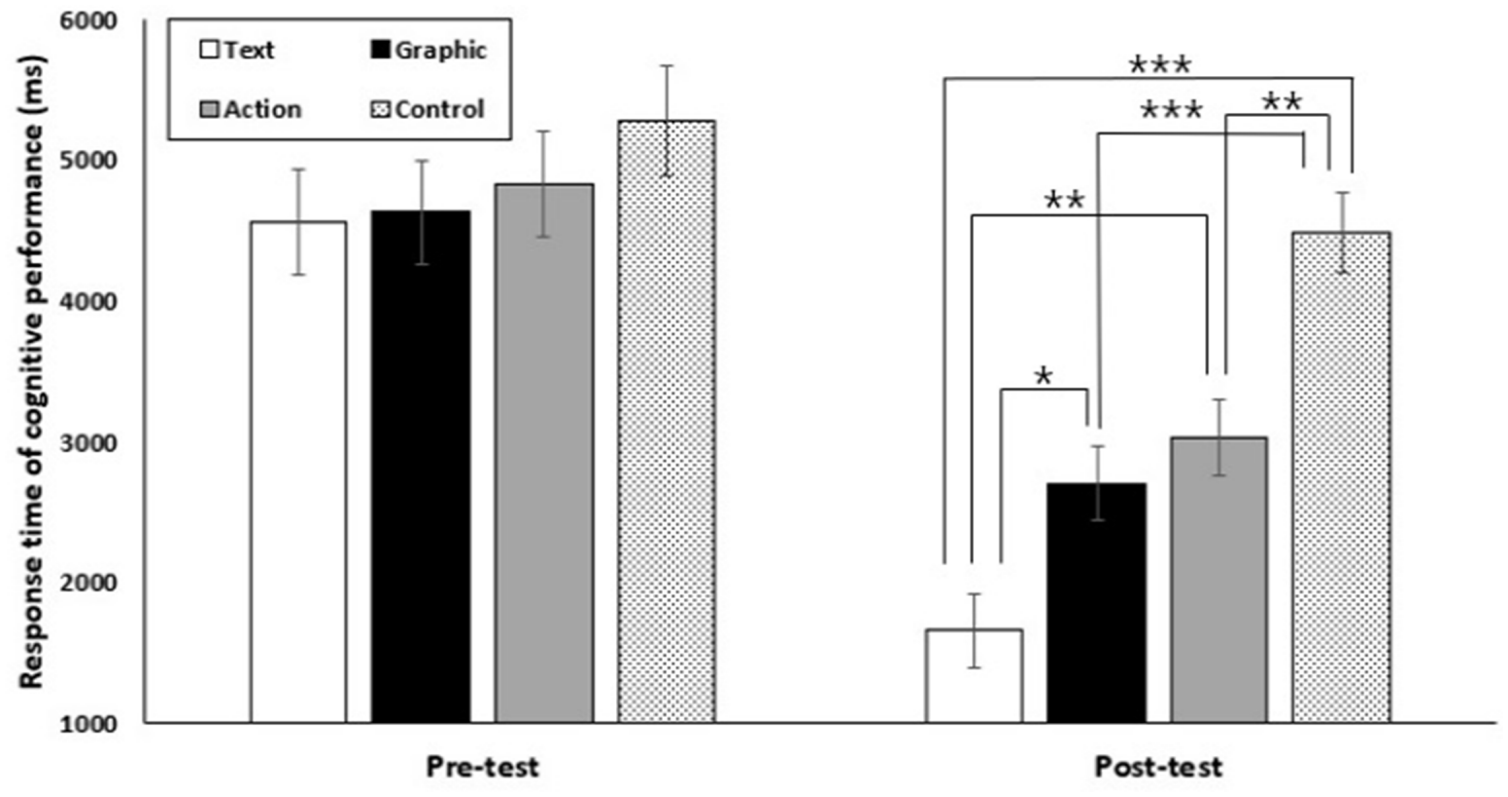
Response time of cognitive performance in working memory for text cue-based action observation (Text), graphic cue-based action observation (Graphic), action observation (Action), and control groups (Control) across pre-test and post-test conditions. Error bars represent standard deviation of the group means. The asterisks indicate significant differences. ^*^p < 0.05, ^**^p < 0.01, ^***^p < 0.001.

The analysis of the response time of cognitive performance in working memory revealed that the main effect of group [*F*(3,40) = 8.55, *p* < 0.001, $\eta_{p}^{2}$ = 0.39] and test session [*F*(1,40) = 91.55, *p* < 0.001, $\eta_{p}^{2}$ = 0.70] were significant, respectively. The result of the post hoc test on the main effect of group showed that the text cue-based AO group and the graphic cue-based AO group were significantly shorter than the control group (*p* < 0.01). The result of the post hoc test on the main effect of test session showed that the response time of the post-test was significantly shorter than that of the pre-test (*p* < 0.001). The interaction effect of group and test session was also significant [*F*(3,40) = 5.17, *p* < 0.01, $\eta_{p}^{2}$ = 0.28] (see Figure 3). Post hoc comparisons by way of paired t-tests revealed that the text cue-based AO group, the graphic cue-based AO group, and the AO group performed significantly shorter in the post-test than in the pre-test (*p* < 0.001). However, the control group did not show a significant difference between the pre-test and the post-test (*p > 0*.05). Post hoc comparisons by way of one-way ANOVA revealed no significant difference between groups in the pre-test (*p > 0*.05). However, in the post-test, the text cue-based AO group performed significantly shorter than the graphic cue-based AO group (*p* < 0.05), AO group (*p* < 0.01), and control group (*p* < 0.001). The AO group was significantly shorter than the control group (*p* < 0.01). The graphic cue-based AO group was significantly shorter than the control group (*p* < 0.001).

## 4 Discussion

This study aimed to determine whether action observation with visual cues can help performers understand the tactical aspects of complex sports by analyzing the effect of text cue-based action observation and graphic cue-based action observation on the accuracy and speed of cognitive information processing in working memory. We hypothesized that text cue-based action observation and graphic cue-based action observation are efficient perceptual-cognitive techniques for information processing in working memory compared to mere action observation. In addition, the text-based action observation group using keywords can provide a more specific role of emphasizing the structure and relationship between elements necessary for quick understanding of the task during action observation to beginners who lack knowledge of the task. Thus, we also hypothesized that the text cue-based action observation group could perform better than the graphic cue-based action observation group. In this study, although there was no difference in general ability between groups at baseline, short-term perceptual-cognitive action observation training improved cognitive performance in working memory. Action observation, including text cue-based action observation, was especially found to benefit both accuracy and response time, which are the cognitive performance evaluation variables in working memory. These results suggest that graphic cue-based and mere action observations are also practical. However, text cue-based action observation can be a relatively more effective perceptual-cognitive training method in strategic training for beginners.

The difference in each individual’s general memory can affect cognitive performance in working memory (Robinson and Brewer, 2020; Rockstroh and Schweizer, 2001). Therefore, the general memory ability of the participants was first examined before the intervention for each group. It was revealed that there was no difference between the participants in the accuracy and response time of general memory ability according to the word memory measured in this process. These results mean that there was no difference in general memory ability among the participants prior to the intervention of each group. In addition, the difference according to the intervention for each group can be regarded as a training effect of cognitive intervention in this study.

Regarding the accuracy of cognitive performance in working memory, there was no difference between groups in the pre-test. However, in the post-test, the text and graphic cue-based action observation groups had higher cognitive performance accuracy than the mere action observation and control groups. There was no difference between the text cue-based action observation group and the graphic cue-based action observation group. In the case of the mere action observation group, the accuracy was improved compared to the control group that did not receive any treatment. These results indicated that cue-based action observation, regardless of whether it was based on text or graphics, was more effective in improving the accuracy of cognitive performance in working memory than mere action observation. Furthermore, action observation training per se, even if it is mere action observation, can improve the accuracy of novices’ cognitive information processing concerning tactical acquisition. Therefore, despite its short duration of approximately 20 min, action observation training improves the accuracy of cognitive information processing in working memory, and these effects are enhanced when cues are included.

There is a broad consensus that working memory and attention are closely linked (Chun, 2011; Fukuda and Vogel, 2009; Gazzaley and Nobre, 2012; Kiyonaga and Egner, 2014; Zanto and Gazzaley, 2009). The concept of attention as a resource argues that cognitive systems have limited resources that can be used for carrying out attention-demanding processes. Given this connection between working memory and attention as resources, the limited capacity of working memory would reflect a limited resource, allowing attention to function where it is allocated. Skilled learners have high attentional control (Lavie, 2005) and are good at excluding irrelevant content from working memory during action observation (Oberauer et al., 2012), so they can better utilize their working memory capacity. Novices, on the other hand, are likely to struggle in this area. The text cue-based action observation and graphic cue-based action observation likely provided visual cues-keyword-based textual information and symbolic representations-to help direct attention within the learner’s limited cognitive resources. This provision of cues probably contributed to learner’s understanding of the doubles formation skills in badminton. Thus, the results suggest that even with a short duration of action observation training, action observation training with cues can increase the accuracy of cognitive performance in working memory.

There was no difference between the groups in the pre-test in the evaluation of cognitive performance in working memory related to response time. This result shows that the participant’s initial working memory processing abilities in speed were similar to the accuracy variable. However, in the post-test, the response time of the text cue-based action observation groups was shorter than that of the other groups, and the mere action observation group showed a shorter response time than the control group. Additionally, it was found that the graphic cue-based action observation group had a shorter response time than the control group. These results, along with the accuracy variables discussed earlier, suggest that in acquiring tactics related to badminton doubles formations, textual cueing based on keywords can be the most efficient action observation training for novices because it reduces decision-making time in complex sports situations. Somewhat different from the previous accuracy results, the graphic cue-based action observation group reduced response time compared to the control group. However, it did not reduce the participant’s response time compared to the mere action observation group. These results show no difference between graphic cue-based and mere action observation in terms of cognitive decision-making time in the short-term application, indicating that novice learners can benefit from graphic cueing to make accurate decisions when provided with important information. However, graphic cueing may impose some cognitive decision-making time compared to text cueing while making such decisions.

It is likely that learners interpreted the cues differently in the text cue-based action observation and graphic cue-based action observation in terms of the specificity of the cues, even if the additional cues were presented with the same meaning (i.e., text cue-based AO: horizontal line connected to the left and right; graphic cue-based AO: left and right arrows) in the action observation materials in this study. Object-file theory (Kahneman and Treisman, 1984) suggests that when a learner is first exposed to a new object, the spatial information of the stimulus may be processed first, followed by the attribute information. In the case of the textual cue with keywords (i.e., cue presented in the form of a rectangle box), since it contained keywords to facilitate a clear understanding of the formation, it is possible that the cue was first processed as spatial information. Then, the critical information provided in the text was encoded in working memory. However, the graphical cues provided by the arrows were more likely to be perceived by the learners as attributes (e.g., color, identity, etc.) rather than spatial information (Kanwisher and Driver, 1992). There was no difference in cognitive performance accuracy in working memory between the text cue-based action observation and graphic cue-based action observation groups, as learners did not have problems identifying the meaning of the cues during task performance. However, regarding response time, the text cue-based action observation group may be the most efficient at perceiving and recognizing information quickly.

Unlike the graphical cues, it is also possible that the textual keyword cues allowed the performers to silently demonstrate the phonological loop element of their working memory. During the intervention of the actual experiment, video recordings were used to document the experimental process, and it was revealed that some participants in the text cue-based action observation group performed the task while softly whispering the keywords when they were presented as textual cues. A simple mouth movement or whisper can improve the ability to immediately recall the information better than no mouth movement at all (Murray, 1965). Therefore, inner speech may interact with working memory to improve the encoding of new material (Marvel and Desmond, 2012; Ravizza et al., 2004). In Baddeley’s (2007) view, the linguistic content of phonological loops can more easily evoke responses that include semantic associations and task-related intentions. The content of working memory thus makes it easier to bias attention to parts relevant to the content held in working memory. Nevertheless, the effectiveness of text cue-based action observation and graphic cue-based action observation in assessing cognitive performance in working memory concerning accuracy and response time was demonstrated compared to the control group, suggesting that cued action observation is strongly needed to strengthen the perceptual-cognitive component of action observation.

The success and duration of working memory retention depend on attention (Fougine, 2008), task type and complexity (Luria et al., 2010), and participants’ internal working memory capacity (Miller, 1956). Accordingly, if there is a difference in participants’ mental effort or perceived task difficulty when performing the task, the results of this experiment may be different. However, there was no significant difference between the groups in the mental effort score and perceived difficulty, indicators of cognitive load investment in the task. These results show that even if the perceptual-cognitive action observation training consisted of a short time applied in this experiment, action observation improves cognitive performance in working memory. In particular, it suggests that text cue-based action observation training methods, including keywords, can be necessary to improve the novice’s understanding of strategic training.

## 5 Conclusion

Taken together, the results of the present experiment suggest that cue-based action observation (i.e., text cue-based action observation in particular) may have an advantage over mere action observation in modulating cognitive load in working memory, and thus cue-based action observation may be a more effective tactical method for the perceptual-cognitive construction in novices. Action observation training with cues has the potential to shorten the duration of perceptual and cognitive decision-making activities, which increases the likelihood that performers will be able to react accurately and quickly to the target behavior under specific environmental conditions in complex sports situations. Since perception and action are interconnected rather than separate processes, repeated perceptual-cognitive training in similar situations, even if not accompanied by actual action, can be a cognitive intervention to help improve performance accuracy and response time. However, this study has certain limitations that should be acknowledged. One limitation is the reliance on single-item measurement tools to assess mental effort and task difficulty. Although Paas’s (1992) mental effort and the task difficulty scale are reliable and practical, they may not capture the multidimensional nature of these constructs. For example, measures such as the NASA Task Load Index (NASA-TLX; Hart and Staveland, 1988) provide a more comprehensive assessment of cognitive load by examining multiple dimensions, including mental demand, physical demand, temporal demand, effort, frustration, and performance. Alternatively, tools such as the subjective workload assessment technique (Reid and Nygren, 1988) or the cognitive load questionnaire (Leppink et al., 2013) could be employed to assess specific aspects of cognitive and mental workload in greater depth. Future studies should consider utilizing these multidimensional tools to better capture the nuances of mental effort and task difficulty. Moreover, the perceptual-cognitive training process in this study did not measure accuracy and response time related to tactical understanding during actual motor activities. This raises the need for future research to investigate whether decision-making can be improved in real-world performance situations through extended perceptual-cognitive training using cue-based action observation. Analyzing players’ actual sports game recordings following perceptual-cognitive strategic training would provide a more concrete answer to whether perceptual-cognitive skills training that does not involve motor activities leads to improved performance on the field. Additionally, compared to perceptual-cognitive skills training alone, well-designed training that integrates and maintains the coupling between perception, cognition, and action is likely to result in better functional performance. Therefore, applying balanced and well-designed training methods will be essential for achieving practical improvements in the field.

## Data Availability

The original contributions presented in the study are included in the article/supplementary material, further inquiries can be directed to the corresponding author.
